# The complete mitochondrial genome of Asian brown flycatcher *Muscicapa latirostris* (Passeriformes: Muscicapidae)

**DOI:** 10.1080/23802359.2019.1674727

**Published:** 2019-11-08

**Authors:** Xiao Min, Cai-Hong Lu, Tai-Yu Chen, Bin Liu, Chang-Hu Lu

**Affiliations:** College of Biology and the Environment, Nanjing Forestry University, Nanjing, China

**Keywords:** *Muscicapa latirostris*, mitochondrial genome, Asian brown flycatcher, phylogenetic analysis

## Abstract

We report the mitochondrial genome of *Muscicapa latirostris*. The overall base composition of the Asian brown flycatcher mitogenome is 24.31% T, 31.62% C, 29.62% A, and 14.44% G, with an A + T content of 53.93%. The total length of the sequence is 18,026 bp (13 protein-coding genes, 22 transfer RNA genes, 2 ribosomal RNA genes, and 2 control regions). Phylogenetic analysis was performed based on the concatenated nucleotide sequences of cytochrome c oxidase subunit I and cytochrome b using the neighbor-joining method and the Kimura 2-parameter model in MEGA 7.0 with 1000 bootstrap replicates.

The Asian Brown Flycatcher is propagated in Daxing'anling, Hulunbeier League in northeastern China, Daxing'anling in the north of Heilongjiang, Xiaoxing'anling in the northeast, and Wandashan in the east and Changbai Mountain in the east of Jilin Province. During the migration, it passes through the west of Jilin, Liaoning, Hebei, Henan, Shaanxi, and Gansu. The region includes Taiwan, Guangdong, Hong Kong, Guangxi and southern Yunnan, and some winters in Guangdong, Hong Kong, Guangxi, and southern Yunnan. Breed abroad in southern Siberia, from the Yenisei River to the east of Russia, the Far East, North Korea, southern Sakhalin Island and northern Japan, wintering in India, Myanmar, Thailand, Sri Lanka, the Philippines, and the Indonesian archipelago. It mainly inhabits deciduous broad-leaved forests, coniferous and broad-leaved mixed forests and coniferous forests, especially mixed forests and coniferous forests along the mountain streams. It is also found in secondary forests and forest margins in the foothills and plains during the migration season and wintering. Shrubs and farmland are among the small bushes and bamboo bushes.

This study reports, for the first time, the complete mitochondrial genome (mtDNA) sequence of *Muscicapa latirostris*. Samples were collected at the bird circle station of the Jiangsu Dafeng Milu National Natural Reserve (33°05′N, 120°49′E) in October 2018 and after sampling, the specimens (NJFU-201808) were stored in the animal specimens museum of Nanjing Forestry University. The mitogenome of *M. latirostris* is a closed, circular molecule composed of 18,026 bp (GenBank accession no.MK770602). The nucleotide composition is 24.31% T, 31.62% C, 29.62% A, and 14.44% G, with an A + T content of 53.93%. There are 22 transfer RNA genes (tRNA), 13 protein-coding genes (PCGs), 2 ribosomal RNA genes (rRNA), and 2 control regions in the mitogenome and it has a typical, vertebrate mitochondrial gene arrangement (Bernt et al. 2013; Liu et al. 2019).

In this study, phylogenetic analysis of *M. latirostris* and 14 other birds was carried out based on the concatenated nucleotide sequences of cytochrome c oxidase subunit I (COI) and cytochrome b (Cyt b), using the neighbor-joining method and the Kimura 2-parameter model in MEGA 7.0, with 1000 bootstrap replicates (Dickinson 2003; Slack et al. 2007). The mitogenome of *M. latirostris* was genetically closest to that of *Muscicapa sibirica* (Figure 1), which is in accordance with the current morphological classification. (Gonzalez et al. 2009; Sun et al. 2019).

**Figure 1.**
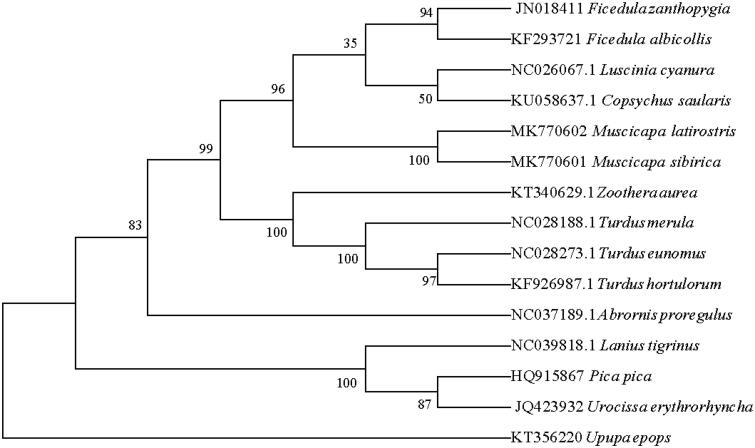
Neighbor-joining phylogenetic tree based on the concatenated nucleotide sequences of cytochrome c oxidase subunit I and cytochrome of *Muscicapa latirostris* and 14 other birds using MEGA 7.0.
